# In Situ Incorporation of TiO_2_@Graphene Oxide (GO) Nanosheets in Polyacrylonitrile (PAN)-Based Membranes Matrix for Ultrafast Protein Separation

**DOI:** 10.3390/membranes13040377

**Published:** 2023-03-26

**Authors:** Wei Zhou, Qiao Liu, Nong Xu, Qing Wang, Long Fan, Qiang Dong

**Affiliations:** 1 Hefei Tianmai Biotechnology Development Co., Ltd., No. 199 Fanhua Ave., Hefei 230601, China; 2 School of Energy, Materials and Chemical Engineering, Hefei University, Hefei 230601, China

**Keywords:** polyacrylonitrile, mixed matrix membrane, graphene oxide, titanium dioxide, ultrafiltration, protein separation

## Abstract

Organic polymeric ultrafiltration (UF) membranes have been widely used in protein separation due to their advantages of high flux and simple manufacturing process. However, due to the hydrophobic nature of the polymer, pure polymeric UF membranes need to be modified or hybrid to increase their flux and anti-fouling performance. In this work, tetrabutyl titanate (TBT) and graphene oxide (GO) were simultaneously added to the polyacrylonitrile (PAN) casting solution to prepare a TiO_2_@GO/PAN hybrid ultrafiltration membrane using a non-solvent induced phase separation (NIPS). During the phase separation process, TBT underwent a sol–gel reaction to generate hydrophilic TiO_2_ nanoparticles in situ. Some of the generated TiO_2_ nanoparticles reacted with the GO through a chelation interaction to form TiO_2_@GO nanocomposites. The resulting TiO_2_@GO nanocomposites had higher hydrophilicity than the GO. They could selectively segregate towards the membrane surface and pore walls through the solvent and non-solvent exchange during the NIPS, significantly improving the membrane’s hydrophilicity. The remaining TiO_2_ nanoparticles were segregated from the membrane matrix to increase the membrane’s porosity. Furthermore, the interaction between the GO and TiO_2_ also restricted the excessive segregation of the TiO_2_ nanoparticles and reduced their losing. The resulting TiO_2_@GO/PAN membrane had a water flux of 1487.6 L·m^−2^·h^−1^ and a bovine serum albumin (BSA) rejection rate of 99.5%, which were much higher than those of the currently available UF membranes. It also exhibited excellent anti-protein fouling performance. Therefore, the prepared TiO_2_@GO/PAN membrane has important practical applications in the field of protein separation.

## 1. Introduction

Protein separation is a critical process in various industries, such as biological pharmacy, genetic engineering, food, and healthcare. It has therefore garnered significant attention from researchers in the field of chemical separation [1,2]. Various techniques have been traditionally used to separate proteins, such as aqueous two-phase extraction [3], gel electrophoresis [4], isoelectric precipitation [5], chromatography [6], crystallization [7], and membrane separation [8]. Among these methods, membrane separation technology is highly efficient and consumes less energy, making it an effective protein treatment method [9,10]. Additionally, it allows easy coupling and avoids destruction of the protein structure, ensuring its integrity and efficacy. Ultrafiltration (UF) is a widely used membrane separation process for protein separation and purification, which relies on size exclusion of contaminants. The mean pore size of the UF membranes is typically in the range of most protein molecules, 1 to 100 nm, making them ideal for protein separation and purification. Polyvinyl difluoride (PVDF) [11], polysulfone (PSf) [12], polyacrylonitrile (PAN) [13], and polyether sulfone (PES) [14] polymers are commonly used due to their excellent processability, physical and chemical resistance, thermal stability, and membrane-forming properties. However, the hydrophobic nature of these polymeric UF membranes often leads to absorption and pore blockage of protein molecules on their top surfaces, known as membrane fouling, which compromises separation performance [15,16]. To address this issue, membrane top surface hydrophilicity, pore size, and porosity can be enhanced to promote protein separation performance. Specifically, a hydrated layer barrier can be constructed on the top surface of a hydrophilic membrane to weaken the interaction between the membrane and protein molecules, inducing limited adsorption on the membrane top surface and in the large pores. Consequently, it will reduce considerable resistance during protein filtration, which will improve membrane separation properties greatly [17,18,19,20,21,22,23]. Various methods have been proposed, including surface grafting [24] and blending [25], to increase membrane top surface hydrophilicity. Among these, incorporating hydrophilic nanoparticles, such as graphene oxide (GO) [26], MXene [27], TiO_2_ [28], and SiO_2_ [29], into polymeric membrane matrices through blending or hybridization is a convenient way to improve membrane hydrophilicity. As a novel two-dimensional (2D) nanomaterial, GO has the characteristic of molecular thickness, abundant hydrophilic chemical groups (–OH, –O–, –COOH) on its surface and edge, as well as high mechanical strength. It has attracted a great deal of attention from researchers. Ganesh et al. dispersed the GO in the PSf membrane matrix and prepared PSf-based mixed matrix membranes (MMMs) using the non-solvent-induced phase separation (NIPS) process. This proved that GO nanosheets can greatly improve membrane hydrophilicity and salt rejection performance [30]. Lee et al. incorporated the GO nanoplates into a polymer matrix to improve the hydrophilicity and anti-fouling properties of a membrane bioreactor (MBR) [31]. Nguyen et al. blended the GO into PSf membrane matrix to enhance its hydrophilic and anti-fouling properties [32]. However, pure GO nanosheets have inadequate hydrophilicity due to their amphiphilic nature with an edge-to-center distribution of hydrophilic to hydrophobic domains [33], which makes it difficult to migrate to the membrane top surface during the NIPS. Hence, pure GO nanosheets were always modified or grafted with chemical groups and hydrophilic nanoparticles to increase their hydrophilicity. Ayyaru et al. prepared sulfonated GO nanosheets to improve PVDF membrane hydrophilicity [34]. The SiO_2_, ZnO, and TiO_2_ nanoparticles were also grafted onto the surface of the GO nanosheets to increase their hydrophilicity [35,36,37]. Moreover, several studies have shown that, including GO nanosheets, many hydrophilic nanoparticles, such as the TiO_2_ and SiO_2_, tend to migrate to the top surface of the polymeric membrane matrix during solvent and non-solvent exchanges of the NIPS, which can greatly improve membrane top surface hydrophilicity and its pore structure [38,39,40]. Generally, the modified GO nanosheets combined with hydrophilic nanoparticles are beneficial for improving the UF performance and anti-protein fouling performance of the membrane.

Herein, TiO_2_@GO nanosheets were synthesized in situ in the PAN membrane matrix during the NIPS formation process. TiO_2_ nanoparticles, generated through the sol–gel process of the precursor, were uniformly grafted onto the top surface of the GO nanosheets. The grafting greatly improved their hydrophilicity. Most of the TiO_2_@GO nanosheets then migrated to the phase interface, including the top surface and pore wall of the membrane, resulting in significant improvements in the membrane’s hydrophilicity, water flux, and anti-fouling properties. Field emission scanning electron microscopy (FESEM), Energy Dispersive X-ray Detector (EDX), Fourier Transform Infrared Spectroscopy (FTIR), and X-ray photoelectron spectroscopy (XPS) were used to characterize the membrane’s morphology, element composition, and chemical structures, respectively. This work demonstrates the successful in situ generation of the TiO_2_@GO nanosheets in the PAN membrane matrix, which holds great potential for promoting the development of the GO-incorporated anti-fouling mixed matrix membranes (MMMs).

## 2. Experimental

### 2.1. Materials

Polyacrylonitrile (Purity ≥ 99.8%, M_W_ = 15 kDa) was purchased from Sigma-Aldrich, Shanghai, China. Flaky graphite (3000 mesh, Purity ≥ 99.5%) was obtained from Qingdao Chenyang Graphite Co., Ltd. (Qingdao, China). Acetic acid glacial (HAc, Purity ≥ 99.0%), N,N-dimethylacetamide (DMAc, Purity ≥ 99.0%), sulfuric acid (H_2_SO_4_, Purity = 95–98%), hydrochloric acid (HCl, Purity = 36–38%), hydrogen peroxide (H_2_O_2_, Purity ≥ 35.0%), KMnO_4_ (Purity ≥ 99.5%), K_2_S_2_O_8_ (Purity ≥ 99.5%), and P_2_O_5_ (Purity ≥ 99.5%) were all purchased from Shanghai Titan Scientific Co., Ltd. (Shanghai, China). Tetrabutyl titanate (TBT, Purity ≥ 99.0%) was obtained from Xilong Chemical Co., Ltd. (Chengdu, China). Bovine serum albumin (BSA, Purity = 96.0–100.0%) was purchased from Newprobo Bio-Tech Co., Ltd. (Shanghai, China). Dialysis bags (MD32, MWCO = 15,000 Da) were purchased from Spectra Medical, Inc (Shanghai, China). Deionized (DI) water was prepared by an ultra-pure water preparation machine (Pall cascada 1, Pall Corporation Shanghai, China) in our lab.

### 2.2. Preparation of the GO

The preparation of the GO nanosheets involved using a modified Hummers’ method [41], in which 325 g flaky graphite was mixed with 15 g K_2_S_2_O_8_, 15 g P_2_O_5_, and 75 mL H_2_SO_4_, and reacted at 80 °C for 4.5 h. The resulting graphite (pretreated powder) was obtained by washing and drying. Subsequently, 300 g dry pretreated powder was mixed with 0.65 L H_2_SO_4_ and 90 g KMnO_4_ at 10 °C. After reacting at 35 °C for 2 h, 1.5 L DI water was added to the mixture with the temperature controlled below 50 °C. Next, the mixture was added to 3 L DI water and stirred for an additional 2 h. Then, 100 mL H_2_O_2_ (30 wt%) was added to the diluted mixture, and a golden suspended solid was obtained. Finally, the suspended solid was filtered, filled into a dialysis bag, immersed in DI water for 7 days, and the resulting GO powder was obtained by freeze-drying.

### 2.3. Preparation of Mixed Matrix Membranes

PAN-based MMMs were fabricated by the NIPS method. Firstly, the GO powder was added to the DMAc and ultrasonically dispersed for 1 h to prepare homogenous GO/DMAc dispersion (0.2 mg/mL). Next, the GO/DMAc dispersion, TBT (0.32 g) and HAc (2 g) were mixed and added dropwise to a PAN/DMAc solution (37.5 g, 16 wt%) under continuous stirring to create a homogenous casting solution. After removing bubbles via vacuum, the casting solution was poured onto a clean glass plate. A thin liquid film was fabricated on the plate with a scraper of 250 μm gap. After waiting for 20 s, the glass plate was immediately immersed in DI water. The resulting membrane, denoted as GO/TiO_2_/PAN, was peeled from the glass plate and stored in fresh DI water for future use. For comparison, pure PAN, GO/PAN, and TiO_2_/PAN membranes were also fabricated using the same procedures without the corresponding components added (as shown in Table 1).

### 2.4. Isolation of the GO in the GO/TiO_2_/PAN Membrane

A piece of dry GO/TiO_2_/PAN membrane was dissolved in the DMAc, producing a turbid solution. The solution was subjected to centrifugal separation using a high-speed centrifuge at 10,000 r/min for 30 min, resulting in a brown solid at the bottom of the centrifuge tube. The solid was then dispersed in the DMAc and DI water through five additional dissolution centrifugal cycles. The resulting centrifugation product was freeze-dried to isolate the GO nanosheets.

### 2.5. Characterization of the Membranes

The viscosity of the casting solution was measured using the NDJ-8S rotational viscometer (Shanghai Pingxuan Scientific Instrument CO., LTD, Shanghai, China) at room temperature.

The morphology of the membrane surfaces and cross-sections was examined using an SEM (FEI-QUANTA 450, Hillsboro, OR, USA) equipped with EDX (Oxford 51-XMX0013, Abingdon, UK) capacity and FESEM (FEI-NOVA NanoSEM 450, USA). To obtain the membrane cross-section, a piece of the membrane was immersed in liquid nitrogen for 5 min and then fractured. To ensure the accurate representation of the membrane structure, five different areas of the same membrane were fractured and observed. Prior to imaging with SEM and FESEM, all samples were gold-sputtered using an EDT-2000 sputter coating instrument (USA).

The chemical structure of the membrane surface was characterized using FTIR (Thermo-Fisher 6700, Waltham, MA, USA) and XPS (Thermo-Fisher ESCALAB^TM^ 250Xi, USA).

The hydrophilicity of the membranes was assessed with a pure water static contact angle apparatus (JC2000D, PowerEach, Shanghai, China). To minimize measurement error, the experiment was replicated five times to obtain the average results.

The overall porosity (ε) was determined using a gravimetric method, which is defined in Equation (1) [42]:

(1) $$\varepsilon =\frac{m_{1}-m_{2}}{Ad\rho }$$

wherein $m_{1}$ is the weight of the wet membrane (g); $m_{2}$ is the weight of the dry membrane (g); A is the membrane area (mm^2^). ρ is the water density (0.998 g/cm^3^), and d is the membrane thickness (mm). The membrane thickness was measured using a digital micrometer.

A Thermal Gravimetric Analyzer (TGA, NETZSCH TG-209F, Selb, Germany) was used to analyze the weight loss of the MMMs in the atmosphere. The heating rate was 10 °C/min, and the final temperature was 900 °C.

A high-speed centrifuge (CENCE, TG16-WS, Changsha, China) was used to separate the GO sheets in the GO/TiO_2_/PAN membrane which was dissolved in the DMAc.

### 2.6. Filtration and Anti-Fouling Tests

A homemade dead-end filtration equipment (Figure S1) was used to characterize the flux and rejection rate of the membrane. The detailed operating steps were as follows: A piece of round wet membrane with radius 4.5 cm was placed in the ultrafiltration cup. The feed solution, DI water or the BSA solution (1 g/L), was then poured into the cup, and the pressure was slowly decreased from 0.15 MPa (which was initially applied for 5 min using a nitrogen cylinder) to 0.1 MPa for testing. The flux of the membrane was measured and calculated with Equation (2):

(2) $$J_{w}=Q/\left(A∆t\right)$$

where $J_{w}$ is the flux (L·m^−2^·h^−1^), *Q* is the permeating volume (L), *A* is the permeating area (m^2^), and ∆t is the permeating time (h).

The anti-fouling property of the membrane was measured by a three-steps cyclic experiment under the same pressure [43]. Firstly, DI water was filtered for 50 min (*J*_0_) in Step 1, and *J*_1_ was measured by filtering the BSA solution for another 50 min in Step 2. After a thorough cleaning, the measured flux of DI water was defined as *J*_2_ in Step 3. In the second anti-fouling test, the same three steps were repeated. Based on these parameters, the flux recovery ratio (FRR), total fouling loss (*R_t_*), reversible flux loss (*R_r_*), and irreversible flux loss (*R_ir_*) were calculated as follows:

(3) $$FRR=\left(\frac{J_{2}}{J_{0}}\right)\times 100\%$$

(4) $$R_{t}=\left(1-\frac{J_{1}}{J_{0}}\right)\times 100\%$$

(5) $$R_{r}=\left(\frac{J_{2}-J_{1}}{J_{0}}\right)\times 100\%$$

(6) $$R_{ir}=\left(\frac{{J_{0}-J}_{2}}{J_{0}}\right)\times 100\%$$

## 3. Results and Discussion

### 3.1. Characterization of the Membranes

**Figure 1:**
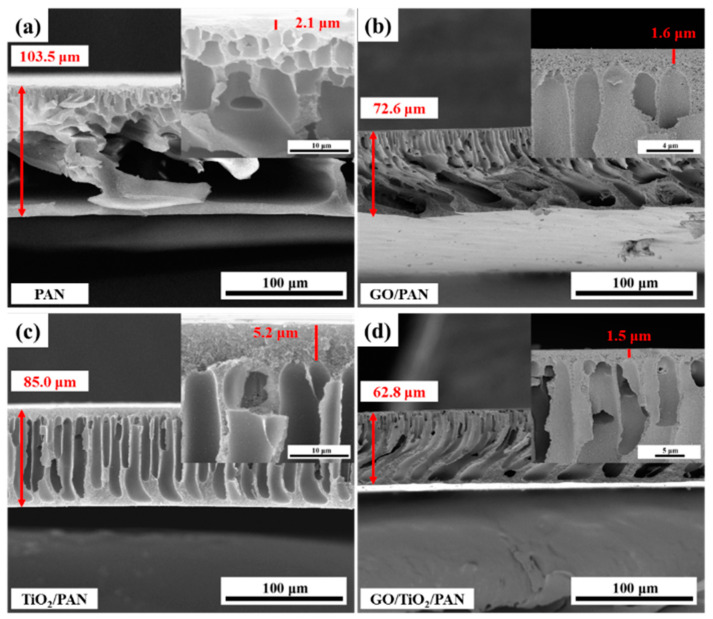
SEM images of the cross-sectional morphologies of the (**a**) PAN, (**b**) GO/PAN, (**c**) TiO_2_/PAN and (**d**) GO/TiO_2_/PAN membranes, and the amplified SEM morphologies of the four membranes’ cross-sections, respectively. The area indicated by the red arrow is the area of membranes thickness measurement.

As shown in Figure 1, a typical asymmetric structure with a dense top skin layer, porous sub-layer, and finger-like support layer can be observed in the cross-section of all four membranes, which originated from the NIPS fabrication [44]. The big hole in the cross-section of the pure PAN membrane demonstrated the fast phase separation process of the PAN/DMAc casting solution in the NIPS, which was due to the hydrophobic nature of the PAN polymer. Finger-like pores were more likely to be observed in the PAN-based MMMs, especially for TiO_2_/PAN membrane (Figure 1c), indicating that the PAN-based casting solution had a lower phase separation speed than the pure PAN casting solution. Maggay et al. demonstrated that a high viscosity of the casting solution slows down the exchange between the solvent and the non-solvent, which helps to maintain the open porous structure and interconnected pores [45]. Therefore, based on the results of viscosity tests (Figure S2), the porous sub-layer structure and straight finger-like pores (Figure 1) in the cross-sections of the PAN-based MMMs mainly originated from the high viscosities of their casting solutions. Furthermore, the interconnected finger-like pores and thin porous sub-layer of the GO/TiO_2_/PAN membrane benefited the protein separation by decreasing the filtration resistance.

**Figure 2:**
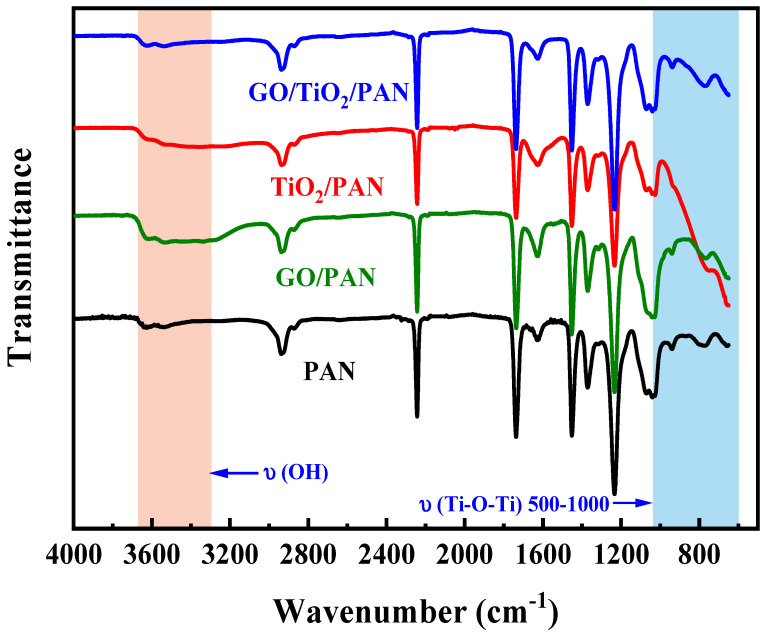
FTIR spectra of the PAN membrane and its MMMs.

Figure 2 shows FTIR spectra of the surface of the pure PAN membrane and its MMMs. The spectra of all the membranes show the specific absorbing peaks of the PAN. The peaks at 2937 cm^−1^, 1451 cm^−1^, and 1370 cm^−1^ were attributed to –C–H stretching modes and in-plane bending vibration of the PAN. The peaks at 2242 cm^−1^, 1233 cm^−1^, and 1736 cm^−1^ were ascribed to –C≡N stretching modes, =C–H in-plane deformation vibration and C=O stretching modes of the PAN, respectively. The peaks at 1071 cm^−1^ and 1040 cm^−1^ were related to C–N stretching modes of the PAN, and the peak at 771 cm^−1^ was ascribed to the skeletal vibration of –(CH_2_)_n_–. The spectra of the GO/PAN, TiO_2_/PAN, and GO/TiO_2_/PAN membranes show peaks with high intensity at wavenumbers ranging from 3200 cm^−1^ to 3700 cm^−1^, which were mainly attributed to the –OH groups originating from the incorporated GO nanosheets (as shown in Figure S3) and TiO_2_ nanoparticles.

Strong peaks at 700 cm^−1^ to 1000 cm^−1^ in the spectra of the TiO_2_/PAN membrane were ascribed to the Ti-O-Ti structure [46], which demonstrates the in situ generation of the TiO_2_ nanoparticles in the TiO_2_/PAN membrane during the NIPS. Due to the exchange of solvent (the DMAc) and non-solvent (DI water) between membrane matrix and coagulation bath, the sol–gel reaction of TBT occurs when it comes into contact with the DI water. This forms TiO_2_ nanoparticles in the membrane matrix [43]. Moreover, what’s particularly interesting is that, despite the same amount of the precursor (TBT) being added to the casting solutions, higher absorption peak intensities at 700 cm^−1^ to 1000 cm^−1^ were observed in the TiO_2_/PAN than in the GO/TiO_2_/PAN membrane.

**Figure 3:**
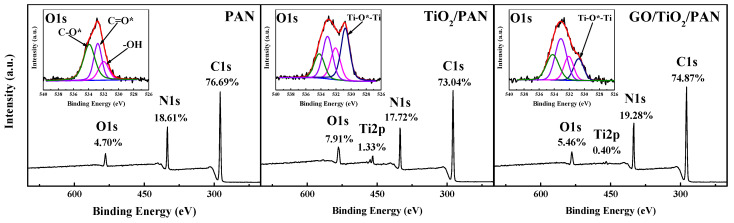
XPS spectra of the PAN, TiO_2_/PAN and GO/TiO_2_/PAN membranes. * indicates the element being analyzed.

As shown in Figure 3, the XPS data of membranes’ top surfaces also indicate that TiO_2_ nanoparticles were observed on the top surfaces of both the TiO_2_/PAN and GO/TiO_2_/PAN membranes (as shown by the Ti–O–Ti peak in the O1s). A higher content of Ti was detected on the top surface of the TiO_2_/PAN membrane (1.33%) than the GO/TiO_2_/PAN membrane (0.40%). Therefore, it is speculated that more TiO_2_ nanoparticles were generated on the top surface of the TiO_2_/PAN membrane than on the GO/TiO_2_/PAN membrane.

To determine the actual amount of the TiO_2_ present in the entire membranes, the burnout characteristics of the TiO_2_/PAN and GO/TiO_2_/PAN membranes were studied by TGA [47]. The TiO_2_/PAN and GO/TiO_2_/PAN membranes were heated in air atmosphere from room temperature to 900 °C at a heating rate of 10 °C/min. The thermal weightlessness of the GO/PAN was 99.4%, indicating that the residue should only be the TiO_2_. Table 2 shows the residual TiO_2_ content in the TiO_2_/PAN and GO/TiO_2_/PAN membranes.

As shown in Table 2, the experimental values and theoretical values of the TiO_2_ in the membranes were listed. The experimental values were determined based on the TGA measurement, and the theoretical values were calculated by the Eq. 7 based on the membrane formation formula assuming complete conversion of TBT into TiO_2_. the actual contents of TiO_2_ were lower than the theoretical contents in both membranes. The actual content of TiO_2_ in the TiO_2_/PAN membrane was higher than that in the GO/TiO_2_/PAN membrane. These results suggested that a portion of the in-situ-synthesized TiO_2_ nanoparticles may be segregated out of the membrane matrix, which was limited by the incorporation of the GO nanosheets. Researchers have long studied the surficial migration behavior of hydrophilic nanoparticles in the polymeric membrane matrix during NIPS. According to the related research [47,48,49], due to the intrinsic characteristics of nanoscale size and high hydrophilicity, in-situ-generated nanoparticles tended to migrate and segregate from the polymeric membrane matrix through the exchange of the solvent and non-solvent. This induced the loss of hydrophilic nanoparticles and was unfavorable for improving the hydrophilicity of the polymeric membranes. In this study, although the incorporated GO nanosheet had high hydrophilicity, its horizontal size of 2 to 3 μm (Figure S4) made it more difficult to segregated from the PAN-based membrane matrix than in-situ-synthesized TiO_2_ nanoparticles due to the geometric size resistance. There might be interactions between the GO nanosheets and TiO_2_ nanoparticles, which limited the excessive segregation of the TiO_2_ nanoparticles. Therefore, the GO nanosheets in the GO/TiO_2_/PAN membrane matrix were isolated for further characterization.

(7) $${TiO}_{2}\left(wt\%\right)=\frac{W_{TBT}\times 23.48\%}{W_{TBT}\times 23.48\%+W_{PAN}+W_{GO}}\times 100\%$$

**Figure 4:**
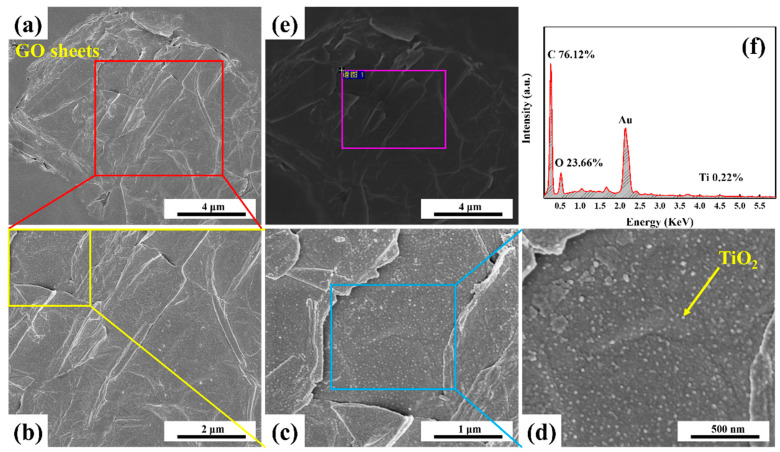
(**a**–**e**) FESEM images of the GO nanosheets which were isolated from the GO/TiO_2_/PAN membranes. (**f**) corresponding EDX spectrum of the GO surface.

As shown in Figure 4a–d, nanoscale particles were uniformly distributed on the surface of the GO nanosheets, whereas the surface of the original GO nanosheets prepared by the modified Hummer’s method was smooth and flat (as shown in Figures S4 and S5). In Figure 4f, Ti was detected on the surfaces of the GO nanosheets, and the C/O value (3.18) was lower than that of the original GO (5.97), indicating that TiO_2_ nanoparticles were synthesized in situ on the surfaces of the GO nanosheets through the sol–gel reaction of TBT during the membrane-forming process of the NIPS. Additionally, the chemical structure of the isolated GO nanosheets were characterized using FTIR.

**Figure 5:**
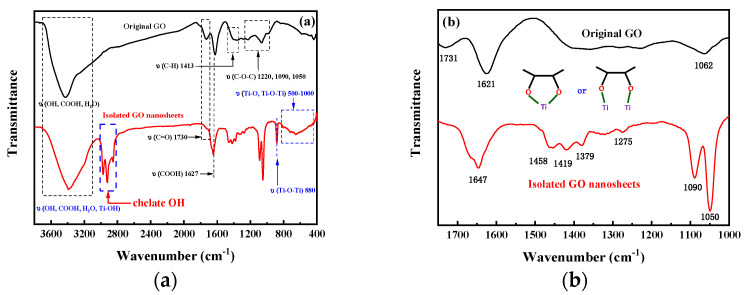
(**a**) the whole and (**b**) partial FTIR spectra of original prepared GO powder and isolated GO nanosheets.

As shown in Figure 5a, the characteristic peaks at 3650 to 3100 cm^−1^, 1730, 1627, and 1413 cm^−1^ were ascribed to –OH, –COOH, and C=O stretching mode vibration of the GO nanosheets, respectively. The peaks at 1220, 1090, and 1050 cm^−1^ were attributed to C–O–C stretching mode vibration of the GO nanosheets. These characteristic peaks were observed in both the GO nanosheets. However, compared to the original GO, characteristic peaks of TiO_2_ at the wavenumber of 800 cm^−1^ and 500 to 1000 cm^−1^ were only observed on the surfaces of the isolated GO nanosheets, indicating the existence of TiO_2_ nanoparticles on the same surface. This finding was consistent with the EDX analysis (Figure 4). Interestingly, characteristic peaks at 1458, 1419, 1379, 1275, and 1090 cm^−1^ were detected on the surfaces of the isolated GO nanosheets (Figure 5b). According to Jankovic et al.’s research, the peaks from the 1090 to 1500 cm^−1^ region were attributed to the conjugate hydroxyl structure between TiO_2_, benzoic acid, and hydroxybenzoic acid, resulting from the chelation of titanium atoms with both phenolic and carboxylic groups [50]. Hence, it is believed that TiO_2_ nanoparticles were bonded to the GO by Ti atoms chelating with hydroxyl and carboxyl groups on the surface and edge of the GO nanosheets.

Based on the above investigation, we can infer that TiO_2_ nanoparticles were synthesized in situ through the sol–gel reaction of TBT during solvent and non-solvent exchanges of the NIPS. Due to the chelation of Ti atoms with hydroxyl and carboxyl groups, the in-situ-generated TiO_2_ nanoparticles were loaded on the surface of the GO nanosheets (denoted as TiO_2_@GO nanosheets). This limited the excessive segregation of TiO_2_ nanoparticles from the PAN-based membrane matrix and benefited to the improvement of membrane hydrophilicity. Additionally, the in situ loading of TiO_2_ nanoparticles resulted in a higher relative content of O element in the TiO_2_@GO nanosheets (Figure 4f) than that of the original GO (Figure S6), indicating an increase in the number of the oxygen-containing functional groups. This enhancement of the hydrophilicity of the GO nanosheets could induce their different migration behaviors in the PAN membrane matrix.

**Figure 6:**
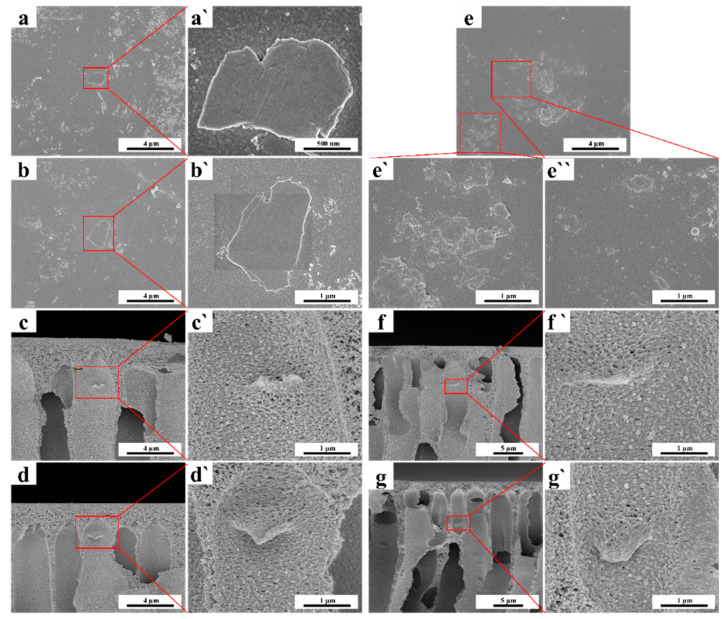
FESEM images of the top surfaces of (**a**), (**a`**), (**b**) and (**b`**) GO/PAN membranes and (**e**), (**e`**) and (**e``**) GO/TiO_2_/PAN membranes, and the cross-sections of (**c**), (**c`**), (**d**) and (**d`**) GO/PAN membranes and (**f**), (**f`**), (**g**) and (**g`**) GO/TiO_2_/PAN membranes.

In Figure 6a–d`, scattered GO nanosheets were observed on the top surface and pore walls of the GO/PAN membrane, indicating that GO nanosheets, along with TiO_2_ nanoparticles and other hydrophilic additives, migrated to the phase interfaces (top surface and pore walls) through the solvent and non-solvent exchanges [38]. Figure 6e–g` show that numerous TiO_2_@GO nanosheets stacked layer-by-layer were observed on the top surface and pore walls of the GO/TiO_2_/PAN membrane. This suggested that high hydrophilicity of the TiO_2_ nanoparticles on the surface can provide a significant driving force for the TiO_2_@GO nanosheets to migrate to the membrane top surface and pore walls. To investigate the distribution of TiO_2_ nanoparticles and GO nanosheets in the membrane matrix, the distributions of Ti, C, and O elements in the membrane cross-section were detected by EDX.

**Figure 7:**
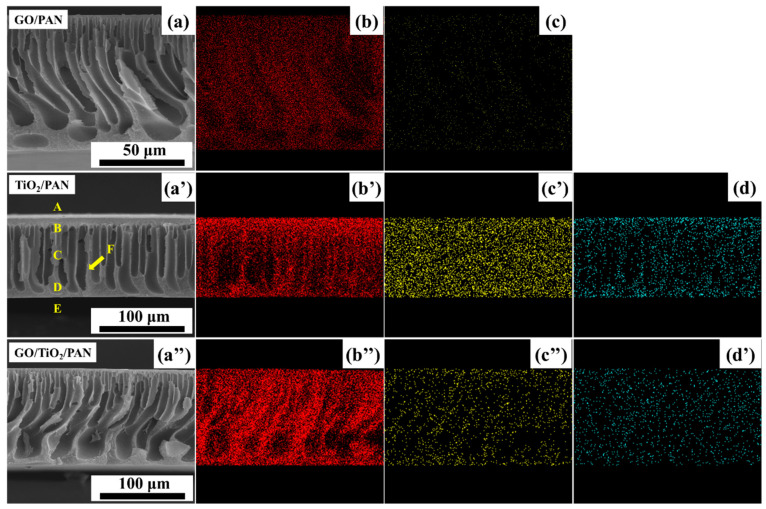
SEM images of the (**a**) GO/PAN, (**a’**) TiO_2_/PAN, and (**a”**) GO/TiO_2_/PAN membranes’ cross-sections. (**b**), (**b**), and (**b”**) element C, (**c**), (**c’**), and (**c”**) element O and (**d**), (**d’**) element Ti distribution (points measured by EDX mapping analysis) on the membranes’ cross-sections. A (top surface), B (upper wall of finger-like pore), C (middle wall of finger-like pore), D (lower wall of finger-like pore), E (bottom surface) and F (middle bulk body) were different regions in the same cross-section.

As shown in Figure 7, there was no obvious aggregation of C, O and Ti elements in the probability plots of the element distributions. This indicates that there was no aggregation of the GO nanosheets, in-situ-generated TiO_2_ nanoparticles, or in situ formed TiO_2_@GO nanocomposites within the PAN-based membrane matrix. The relative contents of C, O, N, and Ti elements in different regions of the membranes were also detected and is shown in Table 3.

As shown in Table 3, the relative contents of C and O in the GO/PAN membrane, O and Ti in the TiO_2_/PAN membrane, and C, O, and Ti in the GO/TiO_2_/PAN membrane were all decreased from region A to F. The elements’ relative contents in the region F for the three MMMs approximated those of the pure PAN membrane (Figure 3), demonstrating that hydrophilic additives, such as the GO nanosheets, TiO_2_ nanoparticles, and the TiO_2_@GO nanocomposites, all had a tendency to migrate towards the phase interfaces of their membranes. However, compared with the GO/PAN and GO/TiO_2_/PAN membranes, more C and O was detected in the GO/TiO_2_/PAN membrane, especially in the regions A and B. This implies that surficial migration happened in most of the TiO_2_@GO nanocomposites because of their high hydrophilicity, which was also consistent with the results of FESEM (Figure 6).

**Figure 8:**
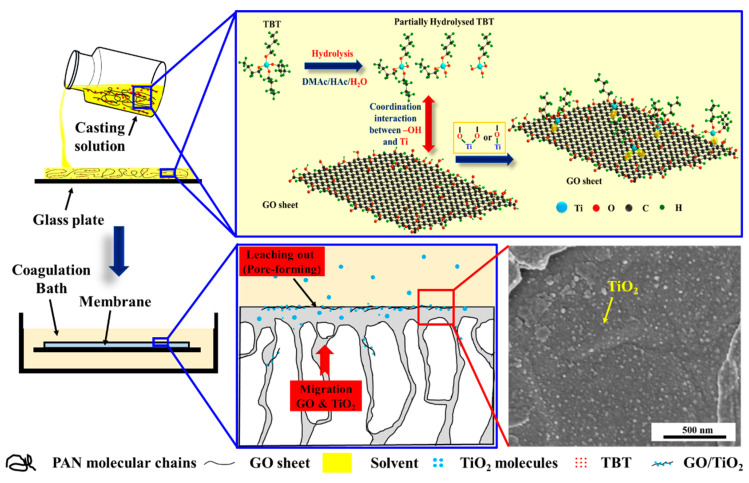
The schematic diagram of the membrane forming mechanism of the GO/TiO_2_/PAN membrane and the synergistic effects between the GO nanosheets and TiO_2_ nanoparticles.

Generally, the in-situ-generated TiO_2_ nanoparticles and the GO nanosheets influenced each other’s surficial migration behavior during the GO/TiO_2_/PAN membrane fabrication. The formation process and mechanism of their interactions are illustrated in Figure 8.

First, a homogenous PAN/TBT/GO/DMAc solution was cast onto a clean glass plate to form a liquid film. Once the film was immersed in DI water, the membrane formation (the NIPS process) occurred. Initially, due to the good intersolubility of the solvent (the DMAc) and non-solvent (water), the DMAc in the liquid film dissolved into water gradually, breaking the thermodynamic equilibrium of the liquid film. This resulted in the differentiation of the original homogenous casting solution into a polymer-rich region and a polymer-poor region [51]. The polymer-rich region mainly comprised the PAN, GO nanosheet, TBT, and a small amount of the DMAc, while most of the DMAc and water were incorporated into the polymer-poor region. The two regions were separated due to the difference in density. The polymer-poor region was gathered in the polymer matrix, creating phase interfaces between the two regions, membrane top surface and pore walls. With the exchange of the DMAc and water between the polymer-poor region and coagulation bath, the water concentration of the polymer-poor region increased, providing opportunities for the contact of the GO nanosheets, TBT, and water. It not only created the conditions for the in situ generation of TiO_2_ nanoparticles but also induced the surficial migration of the GO and TiO_2_ before the polymer-rich region cured in membrane matrix. Importantly, because of the strong chelating interaction between Ti atoms and the GO nanosheets, most of the in-situ-generated TiO_2_ nanoparticles were bonded to the surface of the GO (TiO_2_@GO nanocomposites). This limited the excessive migration of TiO_2_, reduced the loss of the hydrophilic nanoparticles, and promoted the surficial migration of the TiO_2_@GO nanocomposites. According to Chen et al.’s research, physical incompatibilities between the PAN and inorganic materials probably loosened the skin layer of the generated membrane [47]. TiO_2_ nanoparticles with nanoscale size can leach from the top-layer and pore walls into the coagulation bath (pore-forming agent) and can greatly increase membrane porosity [47,52]. However, due to the big horizontal size (Figure S4) and high hydrophilicity, most of the TiO_2_@GO nanocomposites migrate to the top surface and pore walls of the GO/TiO_2_/PAN MMMs, which may greatly enhance the membrane hydrophilicity.

### 3.2. Permeability and Anti-Fouling Properties of the Membranes

**Figure 9:**
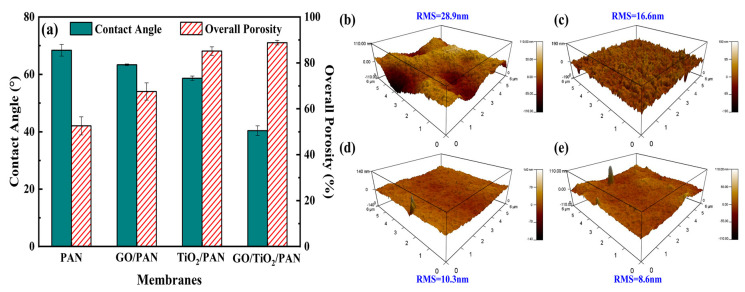
(**a**) contact angle and overall porosity of the PAN, GO/PAN, TiO_2_/PAN, and GO/TiO_2_/PAN MMMs, 3D AFM images and mean roughness of the (**b**) PAN, (**c**) GO/PAN, (**d**) TiO_2_/PAN, and (**e**) GO/TiO_2_/PAN MMMs top surface.

As shown in Figure 9a, the pure PAN membrane had the highest water contact angle (WCA) and the lowest overall porosity among the four tested membranes. The WCA decreased from PAN to GO/TiO_2_/PAN membrane, while the overall porosity increased. The GO/TiO_2_/PAN MMM with WCA of 40.4° and overall porosity of 88.8% had the best hydrophilicity and porous structure among them. Several studies have shown that hydrophilic additives in a polymer matrix lead to faster phase separation [53,54,55]. When combined with the leaching of TiO_2_ nanoparticles, this resulted in a highly porous membrane [38]. Additionally, due to the surficial migration behavior of the GO, TiO_2_, and TiO_2_@GO nanomaterials, the hydrophilicity of the MMMs’ top surfaces was greatly improved, especially that of the TiO_2_@GO nanocomposites in the GO/TiO_2_/PAN MMM. Generally, high porosity reduced the filtration resistance of the membranes, and a hydrophilic membrane top surface resisted the absorption of various protein molecules, which was conducive to enhancing the filtration and anti-fouling performance of all PAN-based membranes.

**Figure 10:**
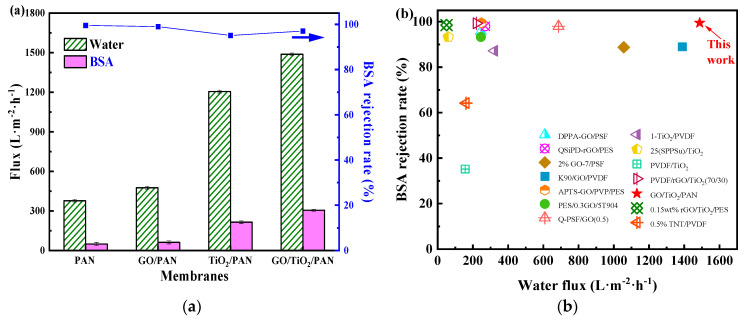
(**a**) Pure water flux and BSA rejection rate of the PAN, GO/PAN, TiO_2_/PAN and GO/TiO_2_/PAN membranes, (**b**) Comparison of the filtration performance in current work with others.

**Figure 11:**
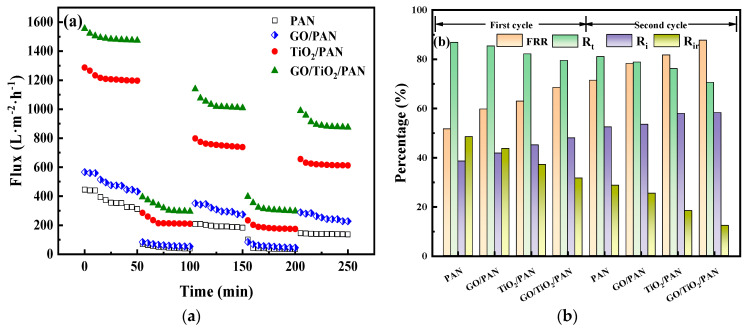
(**a**) Flux variations of the PAN, GO/PAN, TiO_2_/PAN, and GO/TiO_2_/PAN membranes during the three-step filtration cycles with two curves of the BSA results, (**b**) FRR, R_i_, R_ir_, and R_t_ of the PAN, GO/PAN, TiO_2_/PAN, and GO/TiO_2_/PAN membranes in the two filtration circles.

Surface roughness, which greatly influences membrane anti-fouling performance during protein separation, was also detected. In Figure 9b, the pure PAN had the highest mean surface roughness of 28.9 nm. Big “valleys” and “peaks” were observed on the top surface, creating favorable conditions for hiding the protein on the membrane surface. This made it difficult for the absorbed protein to be washed away. However, the surface roughness of all PAN-based MMMs was lower than that of the pure PAN membrane, especially for the GO/TiO_2_/PAN MMM. According to the mechanism of the NIPS process, the rough morphology of the top-layer was mainly attributed to the rapid diffusional exchange of solvent for non-solvent in the top-layer. This led to the vitrification of the maxima of the concentration fluctuations that formed the nodules, namely polymer nodular structure [56,57]. Slow phase separation process of the polymer could limit the generation of polymer nodular and fabricate a smooth top surface of the membrane. [58] From the kinetics perspective, an increase in the viscosity of the casting solution, caused by the addition of the GO nanosheets, TiO_2_ nanoparticles, and TiO_2_@GO nanocomposites (Figure S2), raised the number of molecules per unit volume and restricted the molecules’ motion, resulting in a slowdown of the phase inversion rate. Although many studies have shown that the addition of hydrophilic nanoparticles in the polymeric membrane matrix could increase the membrane surface roughness [59,60,61], the surface roughness of the MMMs was greatly influenced by the aggregation state of the nanoparticles [62,63]. Hence, it could be inferred that the GO nanosheets did not have better distribution state than the in-situ-generated TiO_2_ nanoparticles in the PAN matrix. The TiO_2_ on the surface of the GO could promote dispersion of the TiO_2_@GO nanocomposites, inducing the smooth top surface of the GO/TiO_2_/PAN MMM. The smoother surface always exhibited a lower irreversible attachment of the foulants on the membrane top surface, a higher flux recovery and better anti-fouling property [63,64]. These were analyzed by the BSA rejection and anti-fouling tests as shown in Figure 10 and Figure 11.

In Figure 10a, pure water fluxes of the MMMs were higher than those of the pure PAN membrane (376.9 L·m^−2^·h^−1^). The GO/TiO_2_/PAN membranes, in particular, exhibited a pure water flux of 1487.6 L·m^−2^·h^−1^, which was nearly four times higher than that of the PAN membrane. The pure PAN membrane could reject 99.5% of the BSA molecules in the feeding solution, indicating that the mean pore size of the membrane top surface was less than 8.0 nm and could be classified as a UF membrane [65]. Moreover, although the pure water flux of the MMMs was greatly improved, their BSA rejection rates were still higher than 95%, implying that the mean pore sizes of the membranes top surfaces were not altered by the incorporation of the GO and TiO_2_. Furthermore, the filtration properties of the GO/TiO_2_/PAN MMM were compared with other MMMs mainly incorporating the GO and/or TiO_2_ [26,37,66,67,68,69,70,71,72,73,74,75]. As shown in Figure 10b and Table S1, the fabricated GO/TiO_2_/PAN MMM, with a pure water flux and BSA rejection rate of 1487.6 L·m^−2^·h^−1^ and 99.5%, respectively, had the best UF properties compared with others. Therefore, the GO/TiO_2_/PAN MMM exhibited great potential for application in protein separation.

The anti-fouling performances of the membranes, which were important for the application prospects of the UF membranes, were also analyzed by a three-step cyclic anti-fouling test.

As shown in Figure 11a, the pure water fluxes of the membranes decreased slightly in the first 50 min, likely due to the membrane compaction at 0.15 MPa prior to the measurement. However, when the feed liquid was changed to the BSA solution, the flux decreased sharply because of the deposition and adsorption of the protein on the membrane top surface. After washing, the water flux was still less than the initial value, indicating that some proteins on the membranes’ top surface could not be fully washed away. In the second filtration cycle, the water flux did not reduce significantly, possibly due to the dynamic equilibrium of the absorption and desorption (washing) of the BSA molecules on the membrane surface.

Anti-fouling parameters were calculated using Equations (3)–(6), and the results are presented in Figure 11b and Table S2. It can be seen that the FRR and *R_i_* increased, while the *R_t_* and *R_ir_* decreased, from the PAN, GO/PAN, TiO_2_/PAN, and GO/TiO_2_/PAN membranes in the two anti-fouling cycles. This suggests that the absorbed BSA molecules on the GO/TiO_2_/PAN membrane top surface were more easily washed away than those on the other three. As discussed in Figure 9, the high hydrophilicity and smooth surface of the GO/TiO_2_/PAN membrane ensured that the protein molecules were not easily absorbed on its top surface. The absorbed protein molecules were easily washed away, thereby endowing the membrane with excellent anti-fouling performance.

Moreover, the FRR and *R_i_* were higher and the *R_t_* and *R_ir_* were lower in the second cycle than those in the first cycle, indicating that better anti-fouling performance was exhibited in the second cycle than in the first cycle for the same kinds of membranes. In practice, the adsorption and desorption of the protein molecules on the membranes’ top surfaces were in dynamic equilibrium. Therefore, the anti-fouling parameters in the second cycle could be used to characterize the real anti-fouling performances of the membranes. Compared with other research, the FRR of the GO/TiO_2_/PAN membrane (87.74%) was higher than most of the reported results [13,16,60,71,72,73,74,75,76].

## 4. Conclusions

In this work, inorganic nanoparticle precursor (TBT) and the GO were added to the PAN casting solution to prepare a UF membrane with the TiO_2_@GO/PAN mixed matrix using the NIPS. During the phase separation process, TBT underwent the sol–gel reaction to generate hydrophilic TiO_2_ nanoparticles in situ. Some of the generated TiO_2_ nanoparticles formed the TiO_2_@GO nanocomposites through chelation interactions with the GO. The generated TiO_2_@GO nanocomposites had higher hydrophilicity than the GO, and could bias towards the membrane surface and pore walls through the exchange between the solvents and non-solvents during the NIPS process, significantly improving the hydrophilicity of the membrane. Another part of the TiO_2_ nanoparticles was biased against the membrane body by exchanging the solvents and non-solvents, improving the porosity of the membrane. In addition, the interaction between the GO and TiO_2_ limited the excessive segregation of TiO_2_ nanoparticles, reducing the loss of TiO_2_ nanoparticles. Generally, the interaction between the GO and TiO_2_ significantly improved the hydrophilicity and porosity of the PAN matrix. The water flux and BSA retention of the prepared TiO_2_@GO/PAN MMM were 1487.6 L·m^−2^·h^−1^ and 99.5%, respectively, which was much higher than the performances of current mixed matrix UF membranes. The FRR and *R_t_* were 87.74% and 70.47%, respectively, demonstrating its excellent anti-fouling properties. Moreover, considering the only solvent of BSA molecules, additional work will be done for a fuller characterization of the membrane at a wide range of concentrations, pHs, conductivities, etc. to test the applicability of the current membrane construction to real applications.

## Figures and Tables

**Table 1:** Compositions of pure PAN membrane and the three kinds of PAN MMMs.

Membrane	PAN (g)	DMAc (g)	TBT (g)	GO (mg)	HAc (g)
PAN	6	42	0	0	2
GO/PAN	6	42	0	10.18	2
TiO_2_/PAN	6	41.68	0.32	0	2
GO/TiO_2_/PAN	6	41.68	0.32	10.18	2

**Table 2:** TiO_2_ content in the TiO_2_/PAN and GO/TiO_2_/PAN membranes.

Membranes	TiO_2_ Content in the Membranes (wt%)	
	Experimental Values	Theoretical Values
TiO_2_/PAN	0.62	1.23
GO/TiO_2_/PAN	1.07	1.24

**Table 3:** Relative content of C, O, N, and Ti in different regions of the GO/PAN, TiO_2_/PAN, and GO/TiO_2_/PAN MMMs.

Region	GO/PAN (%)			TiO_2_/PAN (%)				GO/TiO_2_/PAN (%)			
	C	O	N	C	O	N	Ti	C	O	N	Ti
A	77.33	5.12	17.55	73.12	7.88	17.65	1.35	78.71	6.42	14.46	0.55
B	77.08	4.97	17.95	73.44	7.62	17.73	1.21	78.66	6.21	14.62	0.51
C	76.95	4.91	18.14	74.29	6.75	17.93	1.03	78.47	6.07	15.02	0.44
D	76.90	4.88	18.22	76.00	5.12	18.34	0.54	78.16	5.87	15.61	0.36
E	76.87	4.82	18.31	76.40	4.85	18.44	0.31	77.95	5.65	16.12	0.28
F	76.85	4.70	18.45	76.70	4.66	18.52	0.12	77.19	5.13	17.58	0.10

## Supplementary Materials

The following supporting information can be downloaded at: https://www.mdpi.com/article/10.3390/membranes13040377/s1, Figure S1:Schematic diagram of the homemade dead-end filtration equipment in filtration and anti-fouling tests, Figure S2: Casting solutions’ viscosity of the PAN, GO/PAN, TiO_2_/PAN and GO/TiO_2_/PAN membranes, Figure S3: FTIR of the prepared GO nanosheets, Figure S4: AFM images and curves of the horizontal cross sections of the prepared GO nanosheets, Figure S5: TEM images of the prepared GO nanosheets, Figure S6: (a) FESEM images of the original GO nanosheets prepared by the modified Hummer’s method. (b) corresponding EDX spectrum of the GO nanosheet’s surface, Figure S7: XRD curve of the prepared GO nanosheets, Table S1: Comparison of the UF performance with others, Table S2: Anti-fouling parameters of the PAN, GO/PAN, TiO_2_/PAN and GO/TiO_2_/PAN membranes in cyclic anti-fouling tests. References [65,66,67,68,69,70,71,72,73,74,75,76] are cited in the Supplementary Materials.

## Abbreviations

2D	Two-dimensional
BSA	Bovine serum albumin
DI	Deionized
DMAc	N, N-dimethylacetamide
EDX	Energy dispersive X-ray detector
FESEM	Field emission scanning electron microscopy
FRR	Flux recovery ratio
FTIR	Fourier transform infrared spectroscopy
GO	Gaphene oxide
HAc	Acetic acid
MBR	Membrane bio-reactor
MMMs	Mixed matrix membranes
NIPS	Non-solvent induced phase separation
PAN	Polyacrylonitrile
PES	Polyether sulfone
PSf	Polysulfone
PVDF	Polyvinyl difluoride
*R_ir_*	Irreversible flux loss
*R_r_*	Reversible flux loss
*R_t_*	Total fouling loss
TBT	Tetrabutyl titanate
UF	Ultrafiltration
WCA	Water contact angle
XPS	X-ray photoelectron spectroscopy
