# Supplementary material for: In Situ Incorporation of TiO2@Graphene Oxide (GO) Nanosheets in Polyacrylonitrile (PAN)-Based Membranes Matrix for Ultrafast Protein Separation
Source: Membranes (Basel). 2023 Mar 26;13(4):377. doi: 10.3390/membranes13040377 (PMC10142853; doi:10.3390/membranes13040377)
Supplement: Supplementary file 1 [file membranes-13-00377-s001.zip › membranes-2288792-supplementary.pdf]

membranes

MDPI

In Situ Incorporation of TiO_2@Graphene Oxide (GO) Nanosheets in Polyacrylonitrile (PAN)-Based Membranes Matrix for Ultrafast Protein Separation

Wei Zhou [1], Qiao Liu [1,2,*], Nong Xu [2], Qing Wang [2], Long Fan [2] and Qiang Dong [1,2]

[1] Hefei Tianmai Biotechnology Development Co., Ltd., No. 199 Fanhua Ave., Hefei 230601, China
[2] School of Energy, Materials and Chemical Engineering, Hefei University, Hefei 230601, China
* Correspondence: liuqiao@hfuu.edu.cn

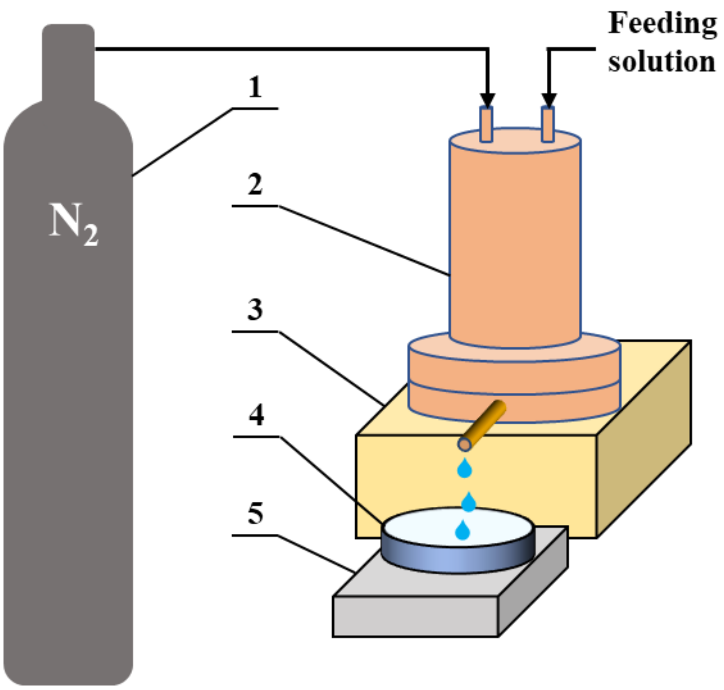

Figure S1. Schematic diagram of the homemade dead-end filtration equipment in filtration and anti-fouling tests. 1. Nitrogen cylinder, 2. Filtration cell, 3. Magnetic Stirrer, 4. Dish, 5. Balance.

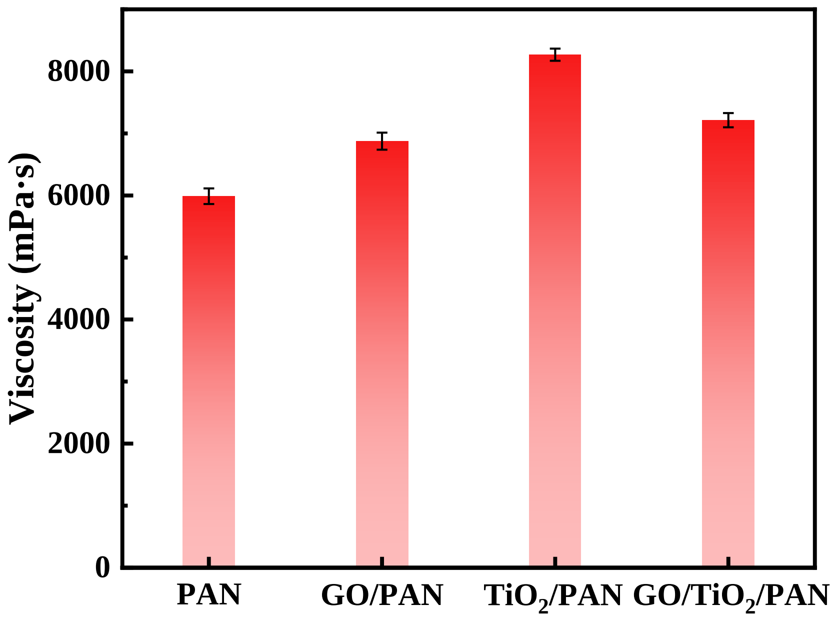

Figure S2. Casting solutions' viscosity of the PAN, GO/PAN, TiO_2/PAN and GO/TiO_2/PAN membranes.

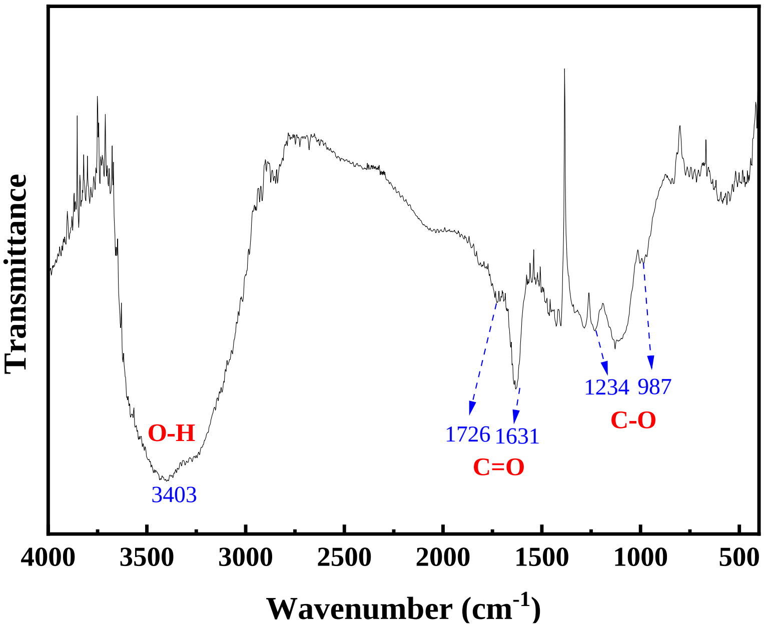

Figure S3. FTIR of the prepared GO nanosheets.

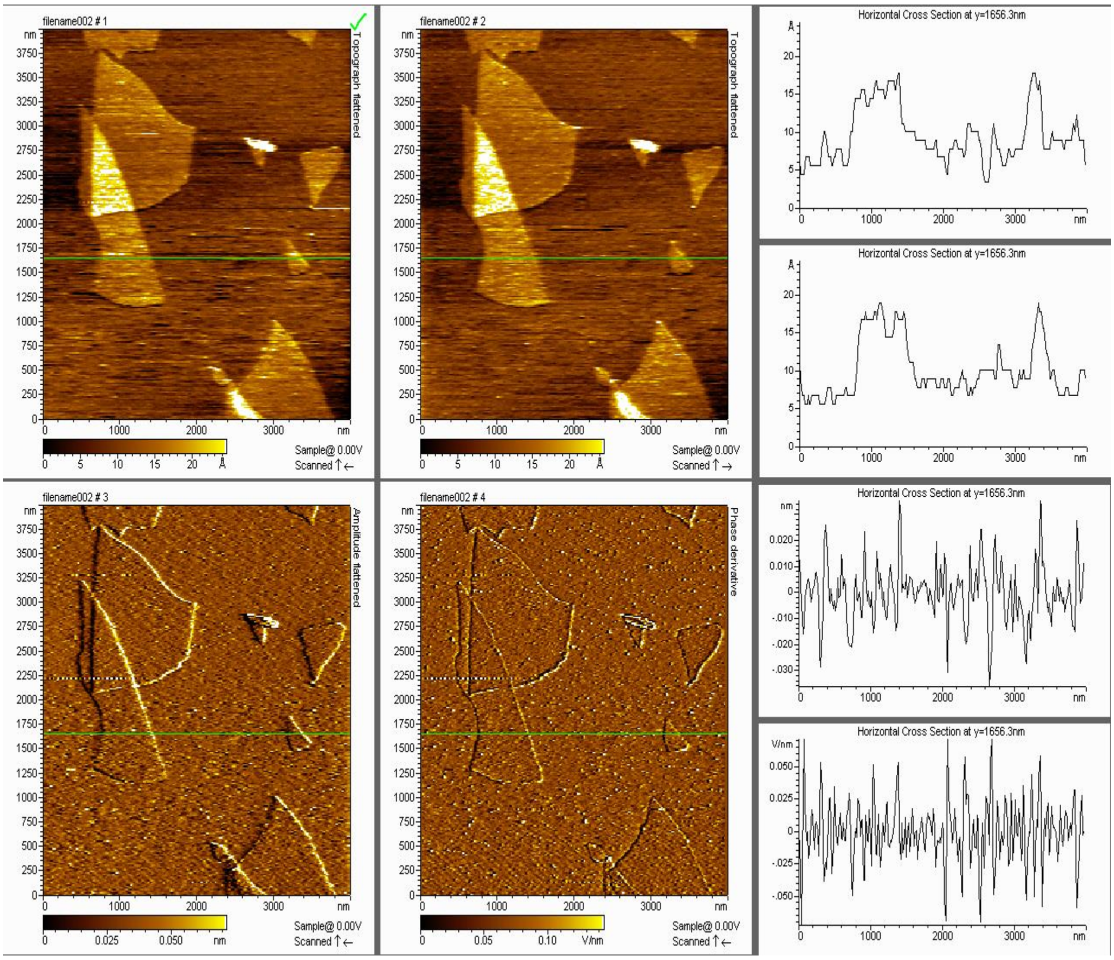

Figure S4. AFM images and curves of the horizontal cross sections of the prepared GO nanosheets.

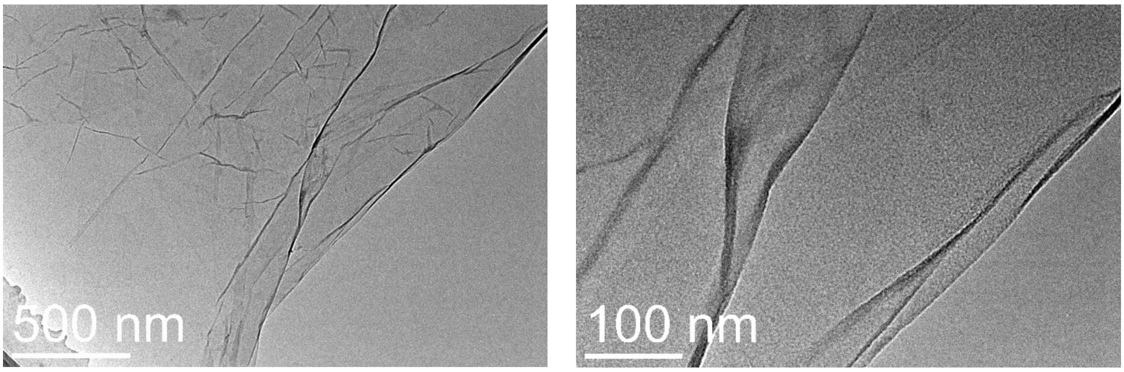

(**a**) (**b**)

Figure S5. (a) and (b) TEM images of the prepared GO nanosheets.

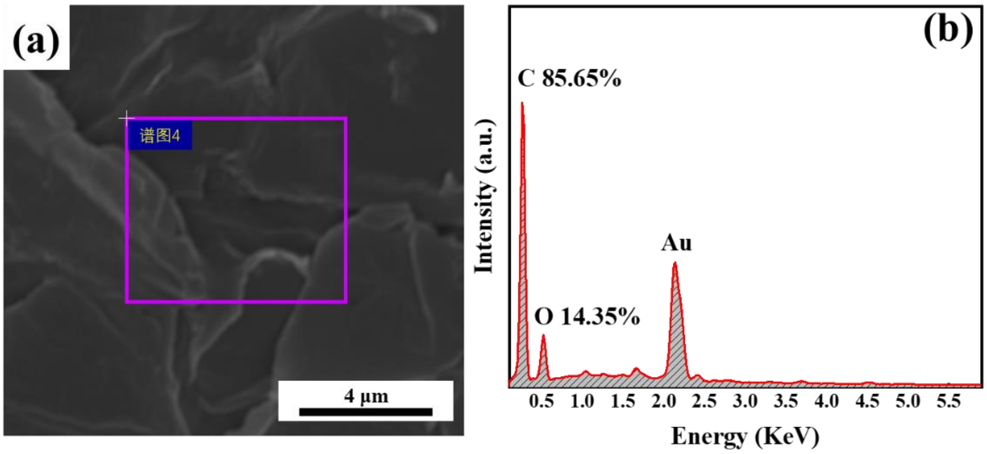

Figure S6. (**a**) FESEM images of the original GO nanosheets prepared by the modified Hummer's method. (**b**) corresponding EDX spectrum of the GO nanosheet's surface.

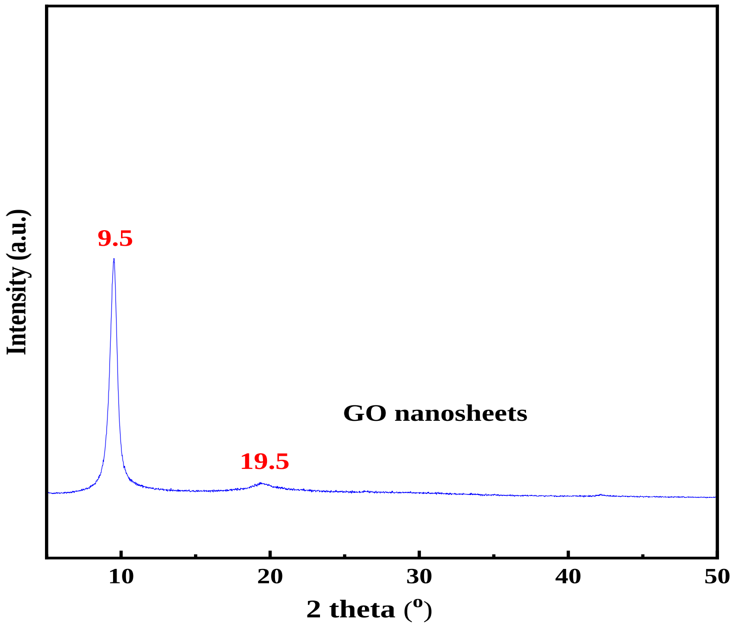

Figure S7. XRD curve of the prepared GO nanosheets.

Table S1. Comparison of the UF performance with others.

Membrane	Symbol	Pressure (MPa)	BSA Concentration ($g \cdot L^{-1}$)	Water Flux ($L \cdot m^{-2} \cdot h^{-1}$)	BSA rejection (%)	Ref.
DPPA-GO/PSF		0.1	0.2	245.1	95.8	[1]
QSiPD-rGO/PES		0.1	0.5	270.0	98.0	[2]
2% GO-7/PSF		0.3	1.0	1056.6	88.7	[3]
K90/GO/PVDF		0.2	0.5	1388.6	89.0	[4]
APTS-GO/PVP/PES		0.4	1.0	248.0	99.5	[5]
PES/0.3GO/5T904		0.1	1.0	245.0	93.3	[6]
Q-PSF/GO(0.5)		0.2	0.5	686.2	98.0	[7]
1-TiO_2/PVDF		0.1	0.5	322.4	87.2	[8]
25(SPPSu)/TiO_2		0.2	0.1	60.0	93.0	[9]
PVDF/TiO_2		0.1	1.0	156.2	35.2	[10]
PVDF/rGO/TiO_2(70/30)		0.3	0.5	219.5	99.3	[11]
0.15wt% rGO/TiO_2/PES		0.5	0.5	50.5	98.5	[12]
0.5%TNT/PVDF		0.1	0.2	160.5	64.2	[13]
GO/TiO_2/PAN		0.1	1.0	1487.6	99.5	This work

Table S2. Anti-fouling parameters of the PAN, GO/PAN, TiO_2/PAN and GO/TiO_2/PAN membranes in cyclic anti-fouling tests.

Membranes	First Cycle				Second Cycle			
	FRR (%)	R_i (%)	R_{ir} (%)	R_t (%)	FRR (%)	R_i (%)	R_{ir} (%)	R_t (%)
PAN	51.62	38.42	48.38	86.80	71.37	52.42	28.63	81.05
GO/PAN	59.67	41.75	43.61	85.36	78.23	53.42	25.38	78.80
TiO_2/PAN	62.89	45.04	37.11	82.15	81.68	57.86	18.30	76.16
GO/TiO_2/PAN	68.43	47.89	31.57	79.46	87.74	58.21	12.26	70.47

References

1. Xu, Z.; Ye, X.; Hu, P.; Min, Y. A.; A, B. L.; Qin, M. B.; A, G. C.; Azido-group functionalized graphene oxide/polysulfone mixed matrix ultrafiltration membrane with enhanced interfacial compatibility for efficient water and wastewater treatment. *Sep. Purif. Technol.* **2022**, *283*, 120162.
2. Ismail, R. A.; Kumar, M.; Thomas, N.; An, A. K.; Arafat, H. A.; Multifunctional hybrid UF membrane from poly (ether sulfone) and quaternized polydopamine anchored reduced graphene oxide nanohybrid for water treatment. *J. Membrane Sci.* **2021**, *639*, 119779.
3. Zambare, R. S.; Dhopte, K. B.; Nemade, P. R.; Tang, C. Y.; Effect of oxidation degree of GO nanosheets on microstructure and performance of polysulfone-GO mixed matrix membranes. *Sep. Purif. Technol.* **2020**, *244*, 116865.
4. Ma, H.; Xie, Q.; Wu, C.; A facile approach to enhance performance of PVDF-matrix nanocomposite membrane via manipulating migration behavior of graphene oxide. *J. Membrane Sci.* **2019**, *590*, 117268.
5. Luque-Alled, J. M.; Abdel-Karim, A.; Alberto, M.; Leaper, S.; Gorgojo, P.; Polyethersulfone membranes: From ultrafiltration to nanofiltration via the incorporation of APTS functionalized-graphene oxide. *Sep. Purif. Technol.* **2020**, *230*, 115836.
6. Abdel-Karim, A.; Leaper, S.; Alberto, M.; Vijayarhavan, A.; Fan, X.; Holmes, S. M.; Souaya, E. R.; Badawy, M.I.; Gorgojo, P.; High flux and fouling resistant flat sheet polyethersulfone membranes incorporated with graphene oxide for ultrafiltration applications. *Chem. Eng. J.* **2018**, *334*, 789–799.
7. Wang, H.; Wang, W.; Wang, L.; Zhao, B.; Zhang, Z. X.; Enhancement of hydrophilicity and the resistance for irreversible fouling of polysulfone (PSF) membrane immobilized with graphene oxide (GO) through chloromethylated and quaternized reaction. *Chem. Eng. J.* **2018**, *334*, 2068–2078.
8. Farahani, M. H. D. A.; Vatanpour, V.; A comprehensive study on the performance and antifouling enhancement of the PVDF mixed matrix membranes by embedding different nanoparticulates: clay, functionalized carbon nanotube, SiO_2 and TiO_2. *Sep. Purif. Technol.* **2018**, *197*, 372–381.

9. Arockiasamy, D. L.; Alhoshan, M.; Alam, J.; Muthumareeswaran.; Figoil, A.; Kumar, A.; Separation of proteins and antifouling properties of polyphenylsulfone based mixed matrix hollow fiber membranes. *Sep. Purif. Technol.* **2017,** *174*, 529–543.
10. Zhang, J.; Wang, Z.; Wang, Q.; Pan, C.; Wu, Z.; Comparison of antifouling behaviours of modified PVDF membranes by TiO_2 sols with different nanoparticle size: Implications of casting solution stability. *J. Membrane Sci.* **2017**, *525,* 378–386.
11. Safarpour, M.; Khataee, A.; Vatanpour, V.; Effect of reduced graphene oxide/TiO_2 nanocomposite with different molar ratios on the performance of PVDF ultrafiltration membranes. *Sep. Purif. Technol.* **2015**, *140*, 32–42.
12. Padaki, M.; Emadzadeh, D.; Masturra, T.; Ismail, A. F.; Antifouling properties of novel PSf and TNT composite membrane and study of effect of the flow direction on membrane washing. *Desalination* **2015**, *362*, 141–150.
13. Subramaniam, M. N.; Goh, P. S.; Lau, W. J.; Tan, Y. H.; Ng, B. C.; Ismail, A. F. Hydrophilic hollow fiber PVDF ultrafiltration membrane incorporated with titanate nanotubes for decolourization of aerobically-treated palm oil mill effluent. *Chem. Eng. J.* **2017**, *316*, 101-110.
